# Should adults with type 2 diabetes be screened for atherosclerotic cardiovascular disease?

**DOI:** 10.12688/f1000research.6625.1

**Published:** 2015-10-28

**Authors:** Yanglu Zhao, Nathan Wong

**Affiliations:** 1 Heart Disease Prevention Program, Division of Cardiology, C240, Medical Sciences, University of California, Irvine, CA, 92697, USA

**Keywords:** cardiovascular disease, non-invasive imaging, asymptomatic diabetes

## Abstract

Diabetes mellitus is associated with greater risks for cardiovascular diseases (CVD). Multiple noninvasive screening tools for CVD including cardiac CT, carotid intima-media thickness test, myocardial perfusion imaging have been examined in those with diabetes, but the prognostic value of these tests vary and issues remain regarding their cost-benefit ratios, potential harms of radiation, and how they fit into screening algorithms for CVD. We discuss in this report the needs and criteria for screening tests and summarize the evidence from observational studies and clinical trials. We also explore whether there should be more sensitive screening modalities to better detect both short and long-term cardiovascular risk among asymptomatic patients with diabetes.

## Introduction

Globally in 2014, 8.3% of the world population or nearly 400 million people had diabetes mellitus (DM) ^1^. Although the prevalence of DM remains high in developed countries, developing countries now comprise the greatest increases in DM prevalence and burden of accompanying comorbidities. Patients with type 2 DM have up to four times the risk of atherosclerotic cardiovascular disease (ASCVD) events compared with those without DM ^2^. Not only do patients with DM have an increased risk of developing ASCVD, but it is often silent ^3, 4^. However, once ASCVD becomes clinically identified, both shorter- and longer-term outcomes in persons with DM are worse than those in persons without DM, and this may be the result of other features associated with DM (e.g., inflammation and prothrombotic tendency). These observations establish the theoretical foundations for the early screening of ASCVD among those with DM. Although the concept for systematic ASCVD screening in DM is appealing, the benefits of such an approach have not been fully demonstrated. As there is ambiguity in outcomes regarding the benefits versus harm of such screening, major organizations, including the American Diabetes Association, currently do not recommend routine screening in those with DM ^5^. Therefore, in this report, we summarize current evidence of pros and cons of screening modalities and propose a framework for screening for subclinical coronary atherosclerosis among asymptomatic patients with DM.

## Need of individualized risk assessment in diabetes

Despite the higher ASCVD risk among persons with diabetes, they are a highly heterogeneous population and diabetes often is not a coronary artery disease (CAD) equivalent. We have previously noted from data in the United States National Health and Nutrition Examination Survey that 32% of men and 48% of women with DM were deemed by the Framingham Risk Score (FRS) to be at low to intermediate risk ^6^. Refining risk estimates in patients with DM may aid in implementing prevention strategies in an efficient and cost-saving manner. Although the Framingham and European risk scores and more recently the American Heart Association/American College of Cardiology (AHA/ACC) Pooled Cohort risk scores emphasize the classic ASCVD risk factors, they are only moderately accurate for the prediction of short- and long-term risk of CVD events ^7^. In addition, the United Kingdom Prospective Diabetes Study (UKPDS) risk engine shows no better performance than FRS and tended to overestimate the coronary heart disease (CHD) risk ^8, 9^. Diabetes in fact can often attenuate the protective effect of optimal levels from other risk factors (dyslipidemia, hypertension, obesity, and so on), and thus the number of traditional risk factors may not be useful in identifying risk in those with DM ^10^. On the other hand, directly examining for subclinical ASCVD, such as by coronary artery calcium (CAC), carotid intima-media thickness (CIMT), endothelial dysfunction, and myocardial ischemia, holds the potential to more accurately discriminate risk in those with DM ^11, 12^.

## Key screening methods for detecting subclinical cardiovascular disease for persons with diabetes mellitus

A good screening test should have the following features: (1) accurately discriminate low- and high-risk persons, (2) produce reliable and reproducible results, (3) provide incremental value to risk predicted by office-based risk assessment, and (4) detect individuals for whom early intervention is likely to have a beneficial impact ^7^. Additional criteria that have been proposed include: (1) ensuring the test identifies a high enough prevalence of disease so that a reasonable number of persons can be identified for intervention and (2) exhibiting high cost-effectiveness ^13^. Currently used modalities may not satisfy all of the criteria completely and instead may vary in providing support for each criterion, thus warranting more studies to provide further validation.

### Coronary artery calcium screening

CAC assesses the extent of calcified atherosclerotic plaques in the coronary arteries and is exquisitely sensitive for detection of atherosclerosis ^14^. CAC scanning has emerged as the most powerful tool for refining risk assessment on top of global risk assessment in asymptomatic individuals ^15^. In those subjects with DM, a CAC score of 0 is associated with ASCVD event rates as low as or lower than those of many persons without DM and increasing CAC scores are associated with progressively higher ASCVD event rates; those with DM who have CAC scores of at least 400 have 10-fold greater event rates (CHD incidence of 4% per year) than those with CAC of 0 (38% of our DM subjects) ^11^. Subjects who undergo scanning for CAC also appear to have improved outcomes in terms of improved risk factor control, including blood pressure, low-density lipoprotein cholesterol, and waist circumference, compared with those not scanned ^16^. This observation may be explained by greater adherence to lifestyle modifications and medical therapy on the basis of visualizing their disease ^17– 20^. Also, CAC screening has been noted to be more cost-effective than myocardial perfusion imaging (MPI); it was estimated that CAC scanning can prevent one event at a cost of $71,249, about a third of the cost of MPI and half that of no screening (treating everyone) ^21^.

Most guidelines have suggested that CAC be considered for screening and risk stratification of patients with DM. Both the 2010 AHA risk assessment guidelines (level IIa) and the 2014 position statement of the Brazilian Diabetes Society (level A) recommend CAC scanning for those who are at intermediate risk or who have diabetes ^22, 23^. The 2012 American Association of Clinical Endocrinology (AACE) Lipid Management Guideline also stated that CAC can be used in those with DM to refine risk stratification and the need for more aggressive preventive strategies ^24^. Most recently, the ACC/AHA guideline on the assessment of cardiovascular risk ^25^ identifies CAC screening (as well as family history of premature ASCVD, ankle brachial index, and high-sensitivity C-reactive protein) as a tool that can be used when, after quantitative risk assessment, a risk-based treatment decision is uncertain. Although current guidelines recommend that all DM patients who are 40 or over be on statin therapy, the intensity of therapy (or possible consideration of therapy in those younger than 40 years of age) may be guided by the use of such testing.

### Stress myocardial perfusion imaging

Observational investigations of MPI have shown high sensitivity (86%) in those with DM and even higher sensitivity among those at higher risk (94%) ^26, 27^. However, the Detection of Ischemia in Asymptomatic Diabetics (DIAD) randomized clinical trial demonstrated that screening patients with DM does not improve clinical outcomes ^28^, even when ischemia is present upon repeat testing (at 3 years) ^29^. The negative result may be due to the low prevalence of CAD and thus a low incidence of coronary events. Although some guidelines have advocated screening for silent myocardial ischemia in high-risk asymptomatic patients with DM ^22^, it is no longer routinely recommended in current guidelines ^5, 23^.

### Coronary computed tomography angiography

Coronary computed tomography angiography (CCTA) allows the evaluation of the full spectrum of CAD from totally normal arteries to non-obstructive disease to significant coronary stenosis and total occlusion. It also allows plaque characterization, including calcified and non-calcified plaque, spotty calcification, positive remodeling, and the napkin ring sign ^30^. Some of these features are associated with a higher likelihood of near-term major adverse cardiovascular events. Several studies have shown the prognostic value of CCTA findings in subjects with asymptomatic DM ^31, 32^. Whether clinical outcomes can be impacted by CCTA screening, however, was only recently reported by the FACTOR-64 randomized clinical trial, which enrolled 900 asymptomatic patients with diabetes; while showing a 20% reduced risk of subsequent adverse cardiovascular events in those screened with CCTA, the findings were not statistically significant and this was due in part to the fairly low event rates ^33^. Due to a lack of sufficient supportive evidence, CCTA is not conventionally recommended for screening asymptomatic individuals with DM in current guidelines ^34– 36^.

### Carotid intima-media thickness

CIMT is an indicator of atherosclerosis in the carotid artery, measuring the combined thickness of the intima and media with B-mode ultrasound. Although CIMT is related to higher CVD event risk ^37^, the meaning of measuring CIMT alone has recently been questioned, as meta-analysis and pooled cohort studies showed that the addition of common CIMT to traditional risk models was associated with only a modest improvement and is unlikely to be of clinical importance ^38, 39^. Similar findings in a cohort of 4,220 patients with DM demonstrated that common CIMT did not add predictive value to the FRS during a median follow-up of 8.7 years ^40^. However, if combined with CAC, ankle brachial index, high-sensitivity C-reactive protein, and family history, the predictive ability for future CHD events may supersede the traditional FRS and UKPDS risk engine ^41^. Importantly, the presence of carotid plaques alone and in combination with CIMT does add to risk prediction beyond FRS, as demonstrated by Nambi and colleagues ^42^, although this question was not specifically evaluated in those with DM in that study.

## What is the future of screening for atherosclerotic cardiovascular disease in diabetes mellitus?

The appropriate use of multimodality screening in those with DM depends on: (1) clinical history of other risk factors, (2) whether the use of a second or third method can address residual risk not addressed by the first method, and (3) determining the correct order to conduct such tests to maximize clinical utility and cost-effectiveness. For instance, CAC scores can accurately reflect the possibility of abnormal stress MPI findings (and with much less radiation), suggesting a role for CAC scoring as a gatekeeper for patients who may benefit from further risk stratification with stress MPI. Expert consensus opinion recommends stress testing imaging in individuals whose CAC score exceeds 400, given that 25% of such subjects will have significant asymptomatic ischemia on MPI. In addition, CAC screening and MPI are complementary for risk assessment since CAC is usually an indicator of anatomic CAD whereas MPI is a physiological test for CAD. Although different algorithms of screening have been proposed, more complete and detailed protocols should be developed and tested for effectiveness to be used in guidelines. Persons with DM are at an increased mortality risk because of CVD, but many receive inadequate treatment for CVD risk factors ^43^. Patients with DM require individualized risk assessment before appropriate intensity of treatment can be implemented. Current screening methods have proven to be effective in predicting future coronary events, but limitations remain in that: (1) few studies concerning the cost-effectiveness of various scanning modalities have been carried out; (2) large randomized clinical trials should be designed to directly look into the impact of screening tests on CVD outcomes as well as the impact on downstream clinical decisions, risk factor changes, and the total medical costs; and (3) few screening methods have directly compared predictive efficacy. The results of such trials will allow us to better identify which screening methods should be employed in patients with DM and help inform therapeutic decision making.
